# Nutritional counseling with or without mobile health technology: a randomized open-label standard-of-care-controlled trial in ALS

**DOI:** 10.1186/s12883-019-1330-6

**Published:** 2019-05-29

**Authors:** Anne Marie Wills, Jamie Garry, Jane Hubbard, Taylor Mezoian, Christopher T. Breen, Courtney Ortiz-Miller, Paige Nalipinski, Stacey Sullivan, James D. Berry, Merit Cudkowicz, Sabrina Paganoni, James Chan, Eric A. Macklin

**Affiliations:** 1 Neurology Clinical Research Institute, Department of Neurology, Massachusetts General Hospital, Boston, MA 02114 USA; 2 Harvard Medical School, Boston, MA USA; 3 Harvard Catalyst/Massachusetts General Hospital Clinical Research Center, Boston, MA USA; 4 Department of Speech, Language, Swallowing and Reading Disabilities, Massachusetts General Hospital, Boston, MA USA; 5 Department of Physical Medicine and Rehabilitation Spaulding Rehabilitation Hospital, Boston, USA; 6 Biostatistics Center, Massachusetts General Hospital, Boston, MA USA

**Keywords:** Amyotrophic lateral sclerosis, ALS, Neurodegenerative disease, Mobile health technology, mHealth, Nutrition, Nutritional counseling, Randomized controlled trial

## Abstract

**Background:**

Nutritional status is an important prognostic factor in Amyotrophic Lateral Sclerosis (ALS). We wished to study the safety, tolerability and efficacy of nutritional counseling with or without an mHealth application to maintain or increase body weight in ALS, compared to standard care.

**Methods:**

In this randomized open-label, standard-of-care-controlled, single-center clinical trial, we randomly assigned adults with ALS to one of three nutritional interventions: counseling by their physician or nurse (“standard care”), counseling by a registered dietitian (RD) (“in-person”), or counseling supported by a mHealth app (“mHealth”). Both intervention arms received tailored nutrition recommendations and recorded dietary intake and weight biweekly (mHealth) or monthly (in-person). The primary outcome of weight and secondary and tertiary outcomes of calorie intake, ALSFRS-R, and quality of life (QOL) were recorded at each clinic visit and analyzed in an ITT mixed model analysis.

**Results:**

A total of 88 participants were enrolled of whom 78 were included in this analysis. The three arms were well-balanced except for excess males in the mHealth arm and greater weight lost at baseline in the in-person arm. Participants in the mHealth arm increased their calorie intake at month 3 to an average of 94% (95% CI: 85, 103) of recommended calories, compared to 81% (95% CI: 72, 91, *p* = 0.06 vs. mHealth) in the standard care arm. After 6 months, calorie intake was not different among the three arms. Overall weight was stable across all three groups. QOL scores in the mHealth arm were stable over 3 months (0.3 points, 95% CI: − 1.7, 2.2) compared to worsening in standard care (− 2.1 points, 95% CI: − 4.0, − 0.2, *p* = 0.09 vs. mHealth), but all scores declined by 6 months. ALSFRS-R total scores declined by an average of − 2.6 points (95% CI: − 5.1, − 0.1) over 6 months in the mHealth arm (*p* = 0.13 vs. standard care) compared to − 5.8 points (95% CI: − 8.2, − 3.4, *p* = 0.74 vs. standard care) in the in-person and − 5.2 points (95% CI: − 7.6, − 2.9) in the standard care arm.

**Conclusions:**

Nutritional counseling by a registered dietitian (with or without support by an mHealth app) is safe but did not maintain weight significantly better than standard care in ALS patients.

**Trial registration:**

https://clinicaltrials.gov/ identifier NCT02418546. Registered April 16, 2015.

**Electronic supplementary material:**

The online version of this article (10.1186/s12883-019-1330-6) contains supplementary material, which is available to authorized users.

## Background

Weight loss is a common symptom of ALS and can occur even before diagnosis [1–3]. Weight loss in ALS is likely due to multiple factors including dysphagia, depression, loss of appetite, difficulty manipulating utensils, and increased energy expenditure due to a hypermetabolic state [4–6]. After diagnosis, body mass index (BMI) is highly correlated with survival [7–9], and moderate obesity has been associated with slower disease progression and longer survival [9, 10]. Weight loss has also been correlated with more rapid disease progression [1, 3], although causation remains uncertain.

We hypothesized that a nutrition intervention to maintain or increase body weight would improve survival in patients with ALS. We previously conducted a phase II multi-center, double-blind, placebo-controlled clinical trial of hypercaloric diets in participants who were receiving percutaneous enteral nutrition [11]. While the study size was small, participants randomized to the high carbohydrate/hypercaloric diet arm experienced significantly fewer adverse events including death (log-rank *p* = 0.03). The ALSFRS-R score also declined by − 1.07 (95% CI: − 1.71, − 0.42) points per month in the high carbohydrate/hypercaloric arm compared to − 2.17 (95% CI: − 3.24, − 1.10) points per month in the control arm (*p* = 0.07) [11].

Here we follow up this result with a clinical trial of hypercaloric nutrition for patients at an earlier stage of the disease. Oral supplements have been tested in two small clinical trials [12, 13] and are being tested in two ongoing larger studies [14]; however, it is not clear whether oral supplements increase the total calories consumed [15]. Instead, we chose to study the effects of nutritional counseling on dietary intake, weight, and disease progression. Nutritional counseling has been studied in one non-randomized [16] and one small randomized trial of limited nutritional counseling [17], with no difference in the rate of BMI decline over 3 months. Nutrition-based mHealth applications are easy to use, commonly available, and can facilitate frequent reminders and measurements [18, 19]. We designed a pragmatic study of nutritional counseling by a registered dietitian with and without the support of an mHealth app compared to standard nutritional counseling by a nurse or physician within an ALS clinic.

## Methods

### Study design and oversight

The electronic health application to measure outcomes remotely (EAT MORE) clinical trial was an investigator-initiated, phase II, prospective, open-label, standard-of-care-controlled, randomized, single-center clinical trial. The primary aims of the study were to test the feasibility, safety, tolerability and efficacy of a mHealth application to maintain or increase body weight compared to in-person nutritional counseling and compared to standard of care.

### Participants

From May 2015 to July 2017, adults with ALS were recruited from the Massachusetts General Hospital (MGH) ALS multidisciplinary clinic. For participants’ convenience, and to enable generalizability of the results, all study activities were performed at the time of routine clinic visits. All participants provided written informed consent prior to screening procedures. At screening, eligible participants had to be adults patients with a diagnosis of possible, probable, laboratory-supported probable or definite ALS using the revised El Escorial criteria [20], able to comply with the consenting process and trial procedures, and able to pass the MGH swallowing screening tool (MGH-SST) [21] with a swallow screening score of 5 or greater. The exclusion criteria included a history of diabetes or BMI greater than 30 kg/m^2^ with a history of cardiovascular disease or any history of unstable medical or psychiatric illness which, in the investigator’s judgment, would prevent the participant from completing their assessments. .

### Randomization and masking

Participants were randomized 1:1:1 to one of three interventions: counseling by their physician and nurse (standard care) vs. nutritional counseling at each clinic visit (in-person) by a registered dietitian, vs. nutritional counseling supported by an mHealth application. The randomization schedule was developed by the MGH Biostatistics Center in permuted blocks of three. Due to the type of intervention, blinding of participants and researchers was not possible.

### Interventions

All participants met with a registered dietitian at their baseline visit to collect their weight and dietary history using a 24-h recall. Baseline calorie intake was calculated by analyzing the 24-h recall using the Nutrition Data Systems for Research (NDS-R, version 2014) [22]. Caloric recommendations for the two intervention arms were calculated using the ALS Calorie Calculator by Kasarskis et al. [23] modified by an additional 117.5–235 kcal/day depending on baseline BMI and weight history (See Additional file 1: Table S2) with the goal of gaining approximately 0.5–1 kg/month. Participants in both intervention arms received written personalized daily calorie goals, recipes, examples of high calorie foods, and advice how to monitor their weight at home. Participants in the standard care arm received general counseling on balanced nutrition and weight maintenance but did not receive specific dietary goals. Participants in the mHealth arm were prescribed dietary goals through an mhealth app (NuPlanit, Boston, MA) available on iOS devices (iPhone or iPad). If participants did not have an iOS device, they were provided an iPad for the duration of the trial. Participants were instructed to complete 4 days of electronic food records and two home weights every 2 weeks. Participants could use the app more than the minimum requirement. Based upon participants’ weight gain or loss during the study, a research RD with access to app data could modify their dietary recommendations empirically. Participants in the in-person nutritional counseling arm received written goals for calorie intake and the treating RD contacted participants at least monthly to ask them to complete paper food records and to weigh themselves at home. The total duration of the nutrition intervention was 6 months +/− 1 month depending on when participants returned for their scheduled clinic appointments. If participants agreed to long-term follow-up, vital status was verified at the end of the study.

### Outcome measures

The primary efficacy outcome was change in weight. Weight was measured at every in-person visit as part of their routine clinical assessment with an electronic scale, chair scale or Hoyer lift with attached scale. If participants were unable to return to the ALS clinic, we obtained weight data from other sources (e.g., visits to other clinics and hospitals). Home weights were analyzed separately from the primary outcome. All study data was entered and stored on PharmaENGINE, a 21 CFR Part 11-compliant, web-based electronic data capture system.

### Safety and tolerability

Safety and tolerability were co-primary outcomes. Participants were asked about adverse events at every encounter: in-person, by telephone or by email. As a result, adverse events were collected more frequently from the in-person dietary counseling arm. Tolerability of the interventions was calculated as the percent of participants who complied with at least 80% of counseling sessions. Participant engagement was defined as the duration and number of interactions with the in-person RD counselor or mhealth app.

Compliance with dietary counseling was measured as the number of calories consumed compared to the calculated dietary recommendations.

### Secondary outcomes

Dietary intake was measured using 24-h and 4-day food records and analyzed using NDS-R software. In addition, the mHealth arm entered food and beverage intake into the mHealth app every 2 weeks. The number of calories consumed was compared to the ALS Calorie Calculator by Kasarskis et al. [23] and to the standard Harris-Benedict, Mifflin-St Jeor equations [24, 25].

### Tertiary outcomes

The ALSFRS-R and Patient Reported Outcomes Measurement Information System short form (PROMIS SF) v 1.1 Global Health QOL questionnaire were collected as tertiary outcomes at every clinic visit (approximately every 3 months). For participants who were unable to return to the clinic, these questionnaires were administered by telephone [26, 27].

### Statistical analysis

All analyses were performed using data from all participants according to their randomization, following the intention-to-treat principle. Variables were summarized as frequencies and percentages, means and standard deviations, or medians and inter-quartile ranges as appropriate. Categorical and continuous variables were compared at baseline by Chi-squared tests and one-way ANOVA, respectively. Change over time of continuous, longitudinal measures was analyzed using a shared-baseline, mixed effect model with fixed effects for visit and an interaction between post-baseline visit and study arm and with a random slope and intercept for each subject with unstructured covariance. The longitudinal models were adjusted for baseline covariates that were found to be imbalanced between the groups (sex and change in BMI since diagnosis and their interactions with visit). Model estimates were reported for an “average” subject in the study population with a mean sex and mean change in BMI since diagnosis. Estimates are unbiased by loss to follow-up if missing data are predictable from observed trajectories given assumptions of the model. Two-sided *p*-values less than 0.027 were considered statistically significant for comparisons of in-person and mHealth treatments to standard care, correcting for multiple comparisons by Dunnett’s method. Change over time in weight was compared with change over time in ALSFRS-R by Pearson correlation of change scores from baseline to months 3 and 6 among complete cases. All analyses were performed using R version 3.4.3. The target sample size for this study was originally 150 subjects for 80% power to detect a 0.75 kg/month difference in weight change; however, we terminated the study after 80 participants were randomized due to budgetary constraints.

## Results

### Study population

**Fig. 1:**
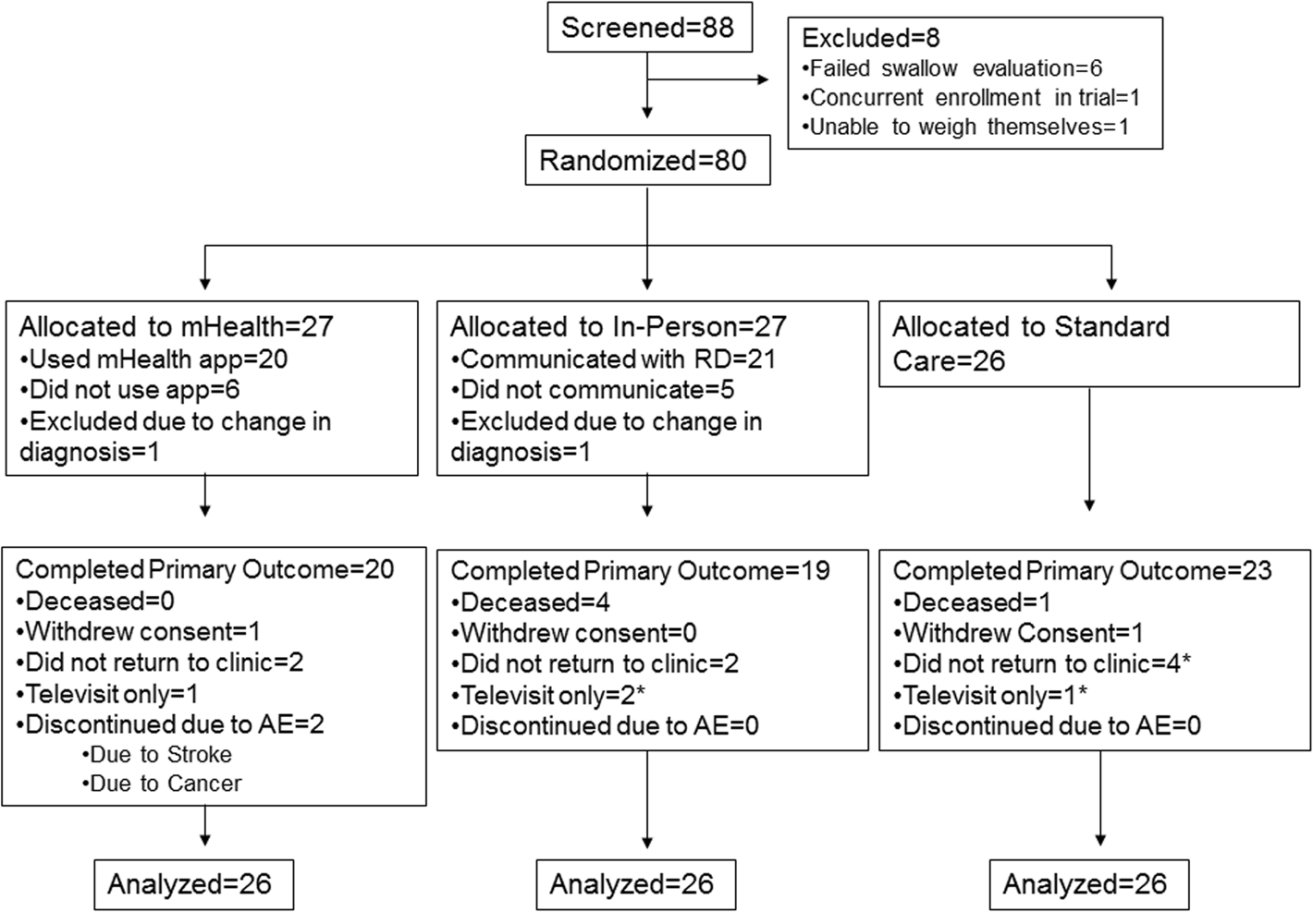
Consort flow diagram. Consort flow diagram showing the final disposition of participants in the study. Participants who did not return to the clinic for their month 6 visit but who supplied weight data for the primary analysis are marked with an asterisk

Eighty-eight people with ALS were screened and 80 participants were randomized. Eight participants were excluded at screening, primarily due to failing the swallow evaluation (Consort diagram, Fig. 1: Enrollment and Outcomes). Two participants were later excluded from the analysis due to a change in diagnosis from ALS. Baseline demographics and disease characteristics were well-balanced across the three arms (Table 1) although participants in the mHealth arm were more likely to be male and participants in the in-person arm had experienced greater weight loss before enrolling in the study. The low number of participants with bulbar onset in all groups was due to the screening swallow evaluation.

**Table 1:** Baseline Characteristics of Participants ^1^

	Standard Care *N* = 26	In-person *N* = 26	mHealth *N* = 26	Overall *p*-value
Male N(%)	11 (42.3%)	16 (61.5%)	20 (76.9%)	0.04
Age (mean ± SD yrs)	57.5 ± 10.9	58.5 ± 11.9	54.7 ± 11.5	0.47
White Not-Hispanic N(%)	24 (92.3%)	25 (96.2%)	25 (96.2%)	0.77
Bulbar Onset N(%)	3 (11.5%)	2 (7.7%)	4 (15.4%)	0.69
ALSFRS-R (mean ± SD)	36.7 ± 5.4	34.9 ± 6.7	37.4 ± 6.2	0.35
Months since symptom onset (mean ± SD)	22.3 (18.0)	26.1 (16.8)	27.1 (18.9)	0.60
BMI (mean ± SD kg/m^2^)	26.8 ± 5.1	25.7 ± 4.1	26.0 ± 4.5	0.70
Weight loss since diagnosis (mean ± SD kg)	3.4 ± 5.5	6.3 ± 7.1	2.1 ± 3.4	0.03
Weight loss since max weight (mean ± SD kg)	7.8 ± 9.4	10.4 ± 10.7	6.0 ± 6.7	0.23
Change in BMI since diagnosis (mean ± SD kg/m^2^)	1.2 ± 1.9	2.2 ± 2.6	0.6 ± 1.0	0.01

^**1**^ Baseline characteristics of participants are shown according to treatment group in frequency (N) and percent or mean and standard deviation (SD); Bulbar onset = symptoms of ALS beginning in the cranial nerves; ALSFRS-R = ALS Functional Rating Scale-Revised; BMI = body mass index

### Primary efficacy outcomes

**Fig. 2:**
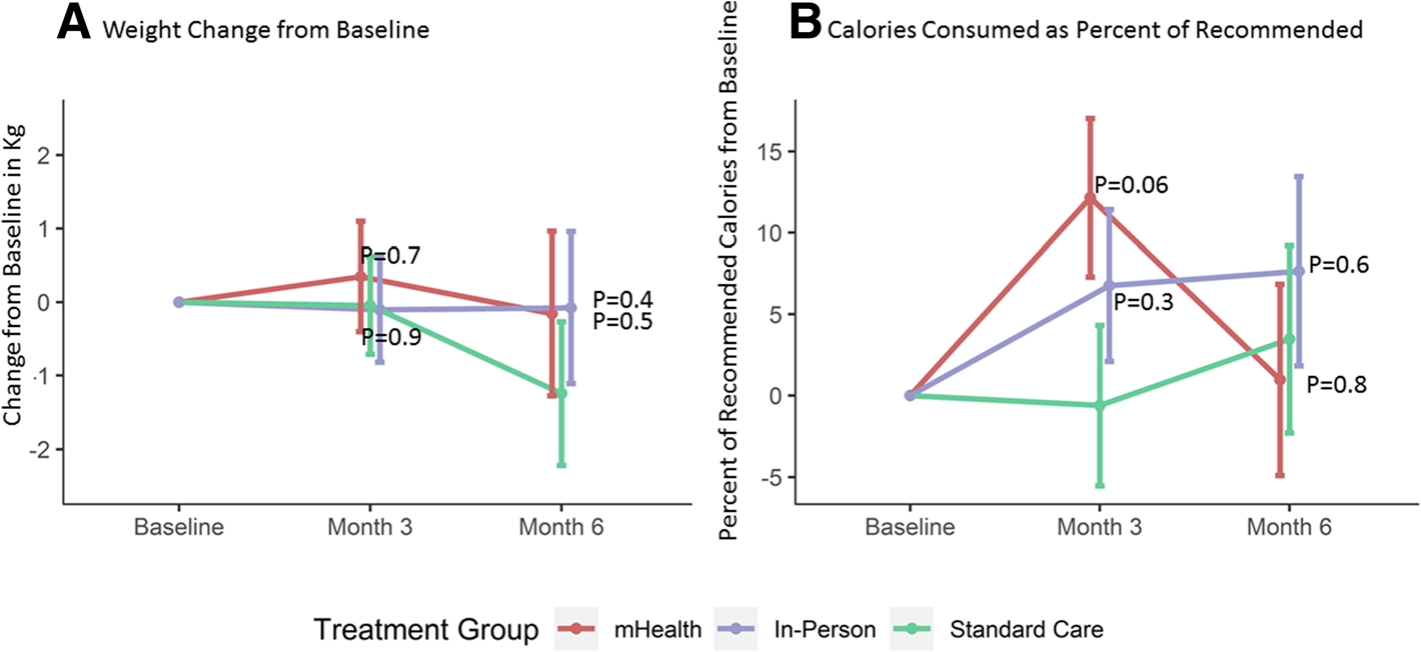
Change in weight and calories consumed by intervention group. **a** Change in weight from baseline by intervention group using measured clinic weights. **b** Calories consumed as a percent of calculated dietary requirements by intervention group at each visit. Red = mHealth; Blue = In-person dietary counseling; Green = Standard Care. Error bars represent 1 standard error around the mean

Eleven percent of participants did not return to the ALS clinic at 3 months and 27% did not return at 6 months, including participants who performed televisits only (see Fig. 1). Alternative sources of data (other hospital and clinic visits) were used to reduce missing 6-month weight data to 23% in the mHealth arm, 27% in the in-person arm and 12% in the standard care arm. Participants in the mHealth arm initially gained an average of 0.3 kg (95% CI: − 1.1, 1.8) at 3 months before losing on average − 0.2 kg (95% CI: − 2.4, 2.1) by 6 months. Participants in the in-person dietary counseling arm lost on average − 0.1 kg (95% CI: − 2.1, 2.0) by 6 months. Participants in the standard care arm lost roughly 1 kg more by 6 months (− 1.2 kg, 95% CI: − 3.2, 0.7) but did not differ statistically from either intervention (*p* = 0.5 and 0.4, respectively; Fig. 2a and Table 2).

**Table 2:** Change over Time in Outcomes by Treatment Group ^1^

				*P*-values	
	Standard Care	In-person	mHealth	In-person vs SC	mHealth vs SC
Change in ALSFRS-R at month 3	−1.9 (− 3.4, − 0.3)	−2.1 (− 3.8, − 0.5)	− 2.0 (− 3.8, − 0.3)	0.80	0.88
Change in ALSFRS-R at month 6	−5.2 (− 7.6, − 2.9)	−5.8 (− 8.2, − 3.4)	− 2.6 (− 5.1, − 0.1)	0.74	0.13
Change in PROMIS QOL at month 3	−2.1 (− 4.0, − 0.2)	− 1.0 (− 2.9, 0.8)	0.3 (− 1.7, 2.2)	0.42	0.08
Change in PROMIS QOL at month 6	−2.8 (− 5.2, − 0.5)	−2.3 (− 4.6, − 0.1)	−1.9 (− 4.4, 0.5)	0.77	0.59
Change in Weight at month 3 (kg)	−0.0 (−1.4, 1.3)	− 0.1 (− 1.5, 1.3)	0.3 (− 1.1, 1.8)	0.95	0.70
Change in Weight at month 6 (kg)	− 1.2 (− 3.2, 0.7)	− 0.1 (− 2.1, 2.0)	−0.2 (− 2.4, 2.1)	0.41	0.47
Change in % Calories at month 3	−0.6 (− 10.4, 9.2)	6.8 (− 2.5, 16.0)	12.2 (2.5, 21.8)	0.26	0.06
Change in % Calories at month 6	3.5 (−7.9, 14.9)	7.6 (−3.9, 19.2)	1.0 (− 10.7, 12.6)	0.59	0.75
Change in total kCal at month 3	−35.3 (− 368.8, 298.1)	250.3 (− 65.2, 565.8)	308.2 (− 21.2, 637.6)	0.21	0.14
Change in total kCal at month 6	83.9 (− 298.7, 466.4)	78.2 (− 295.5, 451.8)	−33.7 (− 424.5, 357.1)	0.98	0.66

^**1**^ Change over time from baseline according to treatment group, adjusted for sex and change in BMI from diagnosis to baseline. Parameter estimates of change from baseline and 95% confidence intervals are shown. ALSFRS-R is shown in units, PROMIS sf v1.1 is shown in units, higher scores are better. Change in % Calories = change in self-reported calories consumed as a percent of recommended calories according to the Kasarskis equation [23]; SC=Standard Care

### Secondary efficacy outcomes

Due to low completion rates for 4-day food records, we used 24-h recalls to measure caloric intake in the three arms. At 3 months, participants in the mHealth arm consumed on average 344 Kcal/day (95% CI: − 118, 805) more than controls (*p* = 0.14) and participants in the in-person arm consumed on average 286 Kcal/day (95% CI: − 160, 731) more than controls (*p* = 0.2) (Table 2, Additional file 2: Figure S1D). Participants in the mHealth and in-person arms experienced similar changes from baseline in macronutrient intake relative to standard care (Additional file 2: Figure S1).

### Tertiary efficacy outcomes

**Fig. 3:**
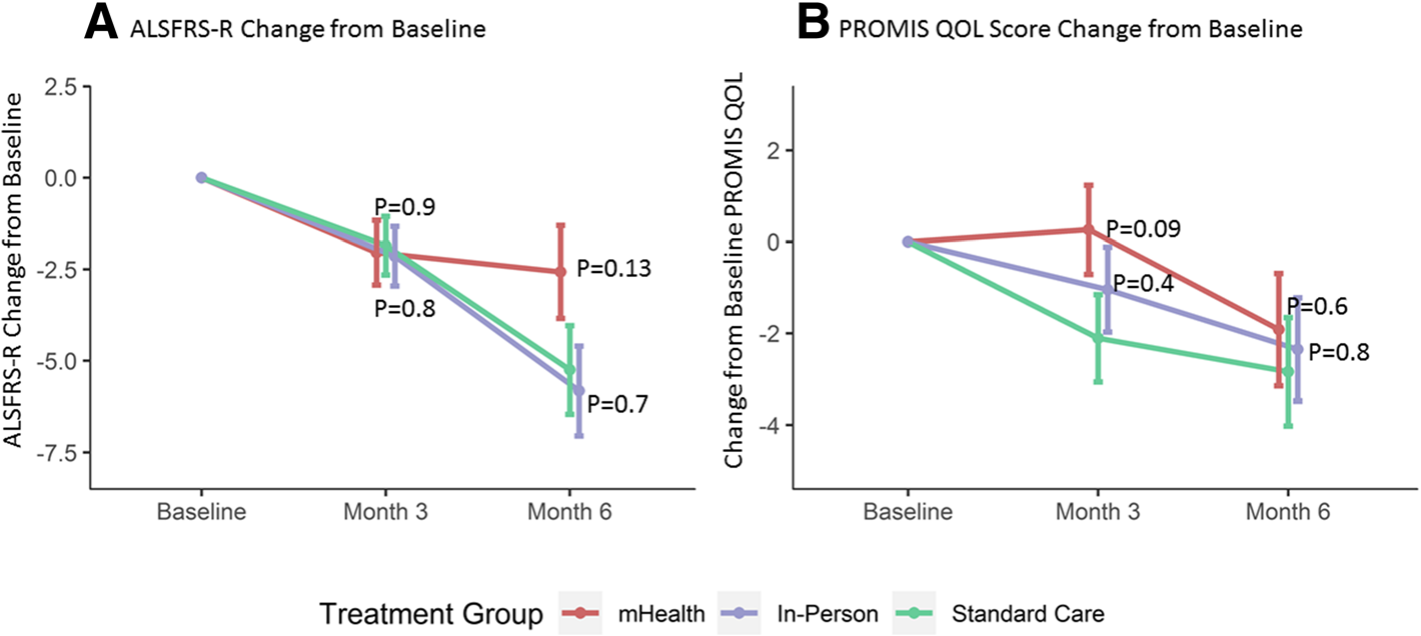
Change in ALSFRS-R and QOL by intervention group. **a** Change in ALSFRS-R from baseline by intervention group. **b** PROMIS SF v 1.1 Global Health QOL scores by intervention group and visit (higher scores are better). Red = mHealth; Blue = In-person dietary counseling; Green = Standard Care. Error bars represent 1 standard error around the mean

While the study was not powered to detect a difference in disease progression, participants in the mHealth arm declined by an average of − 2.6 points (95% CI: − 5.1, − 0.1) on the ALSFRS-R over 6 months compared to − 5.2 points (95% CI: − 7.6, − 2.9) in the standard care arm (*p* = 0.13; Fig. 3a). Participants in the in-person arm also declined by an average of − 5.8 points (95% CI: − 8.2, − 3.4) over 6 months (*p* = 0.7 compared to standard care). Change in weight over 6 months was strongly correlated to the change in ALSFRS-R with a Pearson correlation of r = 0.46 (*p* < 0.001), with greater weight gain associated with slower rates of disease progression.

The 3-month PROMIS QOL scores improved in the mHealth arm by 0.3 units (95% CI: − 1.7, 2.2) while worsening in the standard care arm by − 2.1 units (95% CI: − 4.0, − 0.2; *p* = 0.09; Fig. 3b). The scores also worsened in the in-person arm by − 1.0 units at month 3 (95% CI: − 2.9, 0.8, *p* = 0.4 compared to standard care arm). QOL worsened by month 6 for all participants and were not significantly different among the arms (*p* > 0.5).

### Safety and tolerability outcomes

There were no deaths in the mHealth arm, 1 death in the standard care and 4 deaths in the in-person dietary counseling arm (NS; Additional file 3: Figure S2A). None of the deaths were considered related to the study intervention. Time to placement of a gastrostomy tube was not different between study arms (Additional file 3: Figure S2B).

Adverse events are shown in Additional file 1: Table S3. One participant in the mHealth arm experienced an elevated HgbA1c (7.1%) and had a lacunar stroke after 4 months on the study intervention. There were no other reports of diabetes, stroke, or heart disease during the study.

### Tolerability

In the mHealth arm, 77% of participants entered data into the app and 50% of participants used the app for more than 3 months (Additional file 4: Figure S3). In the in-person arm, 79% of participants engaged with the RD outside of clinic (telephone or email), and 50% maintained at least monthly communication for 6-months. Thus, both interventions failed to meet our a priori criteria for tolerability (compliance with at least 80% of counseling sessions over 6 months). Nutritional counseling was declined by 8% of participants in both intervention arms at month 3 and by 12% of the mHealth and 15% of the in-person participants at month 6. Non-compliance with nutritional counseling did not affect collection of weight or ALSFRS-R as these were collected as part of routine clinical care.

### Compliance with counseling

At baseline, the average estimated calorie intake for all participants was 82% (95% CI: 77, 87%) of their calculated caloric needs. Participants in the mHealth arm increased their intake to an average of 94% (95% CI: 85, 103%) of recommended calories at month 3, compared to 89% (95% CI: 80, 98%) in the in-person arm and 81% (95% CI: 72, 91%) in the standard care arm (*p* = 0.06 for the difference between mHealth and standard care, Fig. 2b). By month 6, calorie intake declined in all three arms.

## Discussion

ALS participants randomized to nutritional counseling with frequent reinforcement by a mobile health app increased their dietary intake and percent of recommended calories compared with participants who received standard of care treatment. While this did not significantly increase weight, the rate of disease progression over 6 months as measured by ALSFRS-R total score was half as fast among mHealth participants compared to participants in the standard care arm. While reverse causation cannot be ruled out, the strong correlation between weight change and change in ALSFRS-R is consistent with our hypothesis that nutritional interventions could be effective in slowing progression of ALS.

The lack of a statistical difference in weight change in either intervention arm may reflect low power due to the smaller than planned sample size and the limited weight loss observed in the standard care arm. Physicians and nurses in our clinic now routinely counsel all ALS patients to eat more calories to avoid weight loss, which likely contaminated our study results. In addition, there was probable contamination of the standard care arm by the consenting process which described the goal of the study as prevention of weight loss. We did not feel that it was ethical to prevent participants from knowing the goal of the study or receiving usual care. It is interesting that an increase in dietary intake during the first 3 months of the intervention did not translate into a significant increase in weight over the same time period. This may be due to the hypermetabolic nature of the disease or due to the short duration of participant engagement in both arms.

Lack of adherence is a common issue with behavioral interventions and mhealth applications in particular, with a reported retention rate for mhealth apps as low as 30% (reviewed in [28, 29]). This may explain, in part, the disappointing results of mhealth apps for treating obesity (reviewed in [28]). It is possible that mhealth apps that engage and monitor participants more frequently and for longer periods would achieve greater weight gain and might slow disease progression more effectively. We believe that reducing the burden of documenting meals and weights should improve adherence.

The lack of efficacy of nutritional counseling alone was surprising and could be due to several factors. First, nutrition counseling sessions were often shortened or canceled to accommodate other multidisciplinary care providers. Second, participants in the mHealth arm were reminded more frequently and had the opportunity to interact with the app more frequently (daily, if desired). Third, participants assigned to the in-person arm had experienced greater weight loss prior to enrollment in the study, although our analyses adjusted for change in BMI prior to enrollment.

There are several limitations to this study. First, the intervention could not be blinded due to the study design; however, the outcomes (weight and ALSFRS-R) were collected in the clinic by staff who were not involved in randomization and were unlikely to be biased by knowledge of study arm allocation. Second, the trial was performed at a single institution, which limits the generalizability of these results. One positive aspect of our single-site design was that the interventions were uniformly administered by the same team of dietitians. A third limitation was the substantial drop-out by month 6. Nevertheless, while participants did not always comply with collection of dietary data, weight and ALSFRS-R were collected as part of routine clinical care, providing more complete outcomes data. We were also interested to note anecdotally that participants who entered the trial with their own devices were more likely to continue to use the app, perhaps reflecting greater comfort with technology and highlighting a potentially important characteristic to control for in future trials of technology-based interventions.

One strength of our study was the enrollment of many participants who would not have qualified for typical ALS clinical trials, including more advanced participants, improving the generalizability of our results. Moreover, our pragmatic study design allowed us to test the real-world effect of having a registered dietitian assigned to the clinic, with realistic follow-up outside the clinic. Our results suggest that more frequent engagement is necessary to achieve dietary goals.

## Conclusions

Our results support the use of this non-pharmacologic treatment for people with ALS. These results are consistent with our prior study which demonstrated that hypercaloric enteral nutrition was associated with improved survival and slower progression. The strong correlation between weight gain and slower ALSFRS-R progression in our study is consistent with the epidemiologic and pre-clinical data. While further study is needed, given the low risk and likely benefit of using an mHealth app to increase calorie intake in ALS, we believe that these methods should be made widely available to people with ALS to help maintain weight.

## Additional files

Additional file 1: **Table S1.** Full Inclusion/Exclusion Criteria. **Table S2.** Weight goals by baseline nutritional status. **Table S3.** Adverse events coded by Medical Dictionary for Regulatory Activities (MedDRA) version 20.0, by system organ class (DOC 111 kb)

Additional file 2: **Figure S1.** Change from baseline in macronutrient intake using 24-h recall data. Supplementary **Figure S1A**. Change from baseline in total carbohydrate intake in grams. Supplementary **Figure S1B**. Change from baseline in total protein intake in grams. Supplementary **Figure S1C**. Change from baseline in total fat intake in grams. Supplementary **Figure S1D**. Change from baseline in total calories (in kCal). Red = mHealth; Blue = In-person dietary counseling; Green = Standard Care. Error bars represent 1 standard error around the mean. (TIF 174 kb)

Additional file 3: **Figure S2A.** Kaplan-Meier survival curves for time to death, permanent assisted ventilation or tracheotomy by treatment group. **Figure S2B.** Time to gastrostomy by treatment group. Red = mHealth; Blue = In-person dietary counseling; Green = Standard Care. The log-rank test result for the difference in survival *p* = 1·0 for the difference between the mHealth group and the standard care group, and *p* = 0·2 for the difference between the in-person counseling group and the standard care group. (TIF 99 kb)

Additional file 4: **Figure S3.** Kaplan-Meier curve for time to last data entry into the mHealth app. Data entry time points were extracted from the mHealth app and used to create a survival curve. (TIF 63 kb)

## Abbreviations

ALS: Amyotrophic Lateral Sclerosis
ALSFRS-R: Amyotrophic Lateral Sclerosis Functional Rating Scale-Revised
CONSORT: Consolidated Standards of Reporting Trials
ITT: Intention to treat
MGH: Massachusetts General Hospital
mHealth: Mobile Health
NDS-R: Nutrition Data Systems for Research
PROMIS: Patient Reported Outcomes Measurement Information System
QOL: Quality of Life
RD: Registered Dietitian
