# Supplementary material for: Nutritional counseling with or without mobile health technology: a randomized open-label standard-of-care-controlled trial in ALS
Source: BMC Neurol. 2019 May 29;19:104. doi: 10.1186/s12883-019-1330-6 (PMC6540456; doi:10.1186/s12883-019-1330-6)
Supplement: Supplementary file 1 — Table S1. Full Inclusion/Exclusion Criteria. Table S2. Weight goals by baseline nutritional status. Table S3. Adverse events coded by Medical Dictionary for Regulatory Activities (MedDRA) version 20.0, by system organ class (DOC 111 kb) [file 12883_2019_1330_MOESM1_ESM.doc]

**Supplementary Table 1: Full Inclusion/Exclusion Criteria**

**4.2 *Subject Inclusion Criteria***

1. Adults with neurodegenerative diseases such as ALS, PD or HD with or without a history of unintentional weight loss.

2. Male or female subjects aged 18 years or older.

3. Participants must be capable of providing informed consent and complying with trial procedures.

4. Participants must have an MGH swallowing screening tool score≥5 at the time of the screening visit [*](#_ENREF_1)

5. Participants or a designated caregiver must be able to obtain weights and communicate to their RD

**4.3 Exclusion criteria:**

1. Clinical evidence of unstable medical or psychiatric illness, in the investigator’s judgment, which would prevent the participant from completing their assessments.

2. BMI > 35 combined with a history of cardiovascular disease; or a history of diabetes regardless of BMI.

* MGH- Swallow Screening Tool [Available from: http://www2.massgeneral.org/stopstroke/swallowScreen.aspx]

**Supplementary Table 2:** Weight goals by baseline nutritional status.

| Baseline BMI | Self-reported weight change since diagnosis | Weight goal during study | Calorie Goals/day |
| --- | --- | --- | --- |
| <25 | Positive or negative | +1 kg/month | Deficit +235 Kcal |
| 25≤30 | <5% of body weight lost | +0.5 kg/month | Deficit +117.5 Kcal |
| 25≤30 | >5% of body weight lost | +1 kg/month | Deficit +235 Kcal |
| 30-35 | <5% of body weight lost | Weight Stability | Deficit +0 Kcal |
| 30-35 | >5% of body weight lost | +0.5 kg/month | Deficit +117.5 Kcal |
| >35 | Positive or Negative | Weight Stability | Deficit + 0 Kcal |

|  | Events | Patients (%) | | Events | Patients (%) | | Events | Patients (%) | | p values | |
| --- | --- | --- | --- | --- | --- | --- | --- | --- | --- | --- | --- |
| **Supplementary Table 3. Adverse Events by System Organ Class** | Standard Care | | | mHealth | | | In-Person | | | mHealth vs SC | In-Person vs SC |
| **Endocrine Disorders (Hypothyroidism)** | 0 | 0 | 0% | 0 | 0 | 0% | 1 | 1 | 4% | p= 1•00 | p= 1•00 |
| **Eye Disorders (Visual Impairment)** | 0 | 0 | 0% | 0 | 0 | 0% | 1 | 1 | 4% | p= 1•00 | p= 1•00 |
| **Gastrointestinal Disorders (Sialorrhea, Dysphagia, Constipation)** | 7 | 7 | 27% | 7 | 5 | 19% | 6 | 4 | 15% | p= 0•74 | p= 0•50 |
| **General Disorders (Fatigue)** | 2 | 2 | 8% | 2 | 2 | 8% | 0 | 0 | 0% | p= 1•00 | p= 0•49 |
| **Infections and Infestations (Upper respiratory, urinary tract infections)** | 1 | 1 | 4% | 2 | 2 | 8% | 0 | 0 | 0% | p= 1•00 | p= 1•00 |
| **Injury, Poisoning and Procedural Complications (Falls, Fractures)** | 17 | 9 | 35% | 15 | 9 | 35% | 8 | 7 | 27% | p= 1•00 | p= 0•76 |
| **Investigations (Hemoglobin Increased, Weight Decreased)** | 2 | 2 | 8% | 1 | 1 | 4% | 0 | 0 | 0% | p= 1•00 | p= 0•49 |
| **Metabolism and Nutritional Disorders (Malnutrition)** | 1 | 1 | 4% | 0 | 0 | 0% | 0 | 0 | 0% | p= 1•00 | p= 1•00 |
| **Musculoskeletal and Connective Tissue Disorders (Muscle Spasms)** | 3 | 3 | 12% | 4 | 3 | 12% | 2 | 2 | 8% | p= 1•00 | p= 1•00 |
| **Neoplasms Benign, Malignant and Unspecified (Ewing's Sarcoma)** | 0 | 0 | 0% | 1 | 1 | 4% | 0 | 0 | 0% | p= 1•00 | p= 1•00 |
| **Nervous System Disorders (Stroke, headache, syncope)** | 4 | 3 | 12% | 4 | 3 | 12% | 0 | 0 | 0% | p= 1•00 | p= 0•24 |
| **Psychiatric Disorders (Insomnia, suicidal ideation)** | 2 | 1 | 4% | 0 | 0 | 0% | 0 | 0 | 0% | p= 1•00 | p= 1•00 |
| **Renal and Urinary Disorders (Urinary incontinence, retention)** | 1 | 1 | 4% | 1 | 1 | 4% | 0 | 0 | 0% | p= 1•00 | p= 1•00 |
| **Respiratory Thoracic and Mediastinal Disorders (Pneumonia, aspiration, pulmonary embolus)** | 10 | 9 | 35% | 2 | 2 | 8% | 3 | 3 | 12% | p= 0•04 | p= 0•10 |
| **Skin and Subcutaneous Tissue Disorders (Decubitus ulcer)** | 1 | 1 | 4% | 0 | 0 | 0% | 0 | 0 | 0% | p= 1•00 | p= 1•00 |
| **Vascular Disorders (Deep venous thrombosis)** | 0 | 0 | 0% | 0 | 0 | 0% | 3 | 3 | 12% | p= 1•00 | p= 0•24 |
| **Serious Adverse Events by System Organ Class** |  |  |  |  |  |  |  |  |  |  |  |
| **Gastrointestinal Disorders (Pancreatitis, Gastroparesis, Dysphagia)** | 0 | 0 | 0% | 3 | 3 | 12% | 0 | 0 | 0% | p= 0•24 | p=1•00 |
| **Neoplasms Benign, Malignant and Unspecified (Ewing's Sarcoma)** | 0 | 0 | 0% | 1 | 1 | 4% | 0 | 0 | 0% | p= 1•00 | p= 1•00 |
| **Nervous System Disorders (Stroke)** | 0 | 0 | 0% | 1 | 1 | 4% | 0 | 0 | 0% | p= 1•00 | p= 1•00 |
| **Respiratory Thoracic and Mediastinal Disorders (Pneumonia, pulmonary embolus)** | 2 | 2 | 8% | 0 | 0 | 0% | 2 | 2 | 8% | p= 0•49 | p= 1•00 |
| **Vascular Disorders (Deep venous thrombosis)** | 0 | 0 | 0% | 0 | 0 | 0% | 2 | 2 | 8% | p= 1•00 | p= 0•49 |

**Supplementary Table 3:** Adverse events coded by Medical Dictionary for Regulatory Activities (MedDRA) version 20.0, by system organ class. Events columns show absolute numbers of adverse events reported in each group. Patients columns show the number of participants who had at least one adverse event (with the % of all patients in that group). Specific adverse events listed in parentheses are a selection of events from all MedDRA verbatim terms reported. SC=Standard Care.

**Checklist of Items for Reporting Trials of Nonpharmacologic Treatments***

| **Section** | **Item** | **Standard CONSORT Description** | **Extension for Nonpharmacologic Trials** | **Reported on Page No.** |
| --- | --- | --- | --- | --- |
| Title and abstract† | 1 | How participants were allocated to interventions (e.g., “random allocation,” “randomized,” or “randomly assigned”) | In the abstract, description of the experimental treatment, comparator, care providers, centers, and blinding status | 1,3 |
| **Introduction** |  |  |  |  |
| Background | 2 | Scientific background and explanation of rationale |  | 5 |
| **Methods** |  |  |  |  |
| Participants† | 3 | Eligibility criteria for participants and the settings and locations where the data were collected | When applicable, eligibility criteria for centers and those performing the interventions | 6 |
| Interventions† | 4 | Precise details of the interventions intended for each group and how and when they were actually administered | Precise details of both the experimental treatment and comparator | 7,8 |
|  | 4A |  | Description of the different components of the interventions and, when applicable, descriptions of the procedure for tailoring the interventions to individual participants | 7,8 |
|  | 4B |  | Details of how the interventions were standardized | 7 |
|  | 4C |  | Details of how adherence of care providers with the protocol was assessed or enhanced | 8 |
| Objectives | 5 | Specific objectives and hypotheses |  | 5 |
| Outcomes | 6 | Clearly defined primary and secondary outcome measures and, when applicable, any methods used to enhance the quality of measurements (e.g., multiple observations, training of assessors) |  | 8,9 |
| Sample size† | 7 | How sample size was determined and, when applicable, explanation of any interim analyses and stopping rules | When applicable, details of whether and how the clustering by care providers or centers was addressed | 10 |
| Randomization–  sequence generation† | 8 | Method used to generate the random allocation sequence, including details of any restriction (e.g., blocking, stratification) | When applicable, how care providers were allocated to each trial group | 7 |
| Allocation concealment | 9 | Method used to implement the random allocation sequence (e.g., numbered containers or central telephone), clarifying whether the sequence was concealed until interventions were assigned |  | 7 |
| Implementation | 10 | Who generated the allocation sequence, who enrolled participants, and who assigned participants to their groups |  | 7 |
| Blinding (masking)† | 11A | Whether or not participants, those administering the interventions, and those assessing the outcomes were blinded to group assignment | Whether or not those administering co-interventions were blinded to group assignment | 7 |
|  | 11B |  | If blinded, method of blinding and description of the similarity of interventions† | n.a. |
| Statistical methods† | 12 | Statistical methods used to compare groups for primary outcome(s); methods for additional analyses, such as subgroup analyses and adjusted analyses | When applicable, details of whether and how the clustering by care providers or centers was addressed | n.a. |
| **Results** |  |  |  |  |
| Participant flow† | 13 | Flow of participants through each stage (a diagram is strongly recommended)---specifically, for each group, report the numbers of participants randomly assigned, receiving intended treatment, completing the study protocol, and analyzed for the primary outcome; describe deviations from study as planned, together with reasons | The number of care providers or centers performing the intervention in each group and the number of patients treated by each care provider or in each center | Figure 1 |
| Implementation of intervention† | New item |  | Details of the experimental treatment and comparator as they were implemented | 7,8 |
| Recruitment | 14 | Dates defining the periods of recruitment and follow-up |  | 6 |
| Baseline data† | 15 | Baseline demographic and clinical characteristics of each group | When applicable, a description of care providers (case volume, qualification, expertise, etc.) and centers (volume) in each group | Table 1 |
| Numbers analyzed | 16 | Number of participants (denominator) in each group included in each analysis and whether analysis was by “intention-to-treat”; state the results in absolute numbers when feasible (e.g., 10/20, not 50%) |  | Figure 1 |
| Outcomes and estimation | 17 | For each primary and secondary outcome, a summary of results for each group and the estimated effect size and its precision (e.g., 95% confidence interval) |  | 11, Table 2 |
| Ancillary analyses | 18 | Address multiplicity by reporting any other analyses performed, including subgroup analyses and adjusted analyses, indicating those prespecified and those exploratory |  | n.a. |
| Adverse events | 19 | All important adverse events or side effects in each intervention group |  | 12, Supplementary Table 3 |
| **Discussion** |  |  |  |  |
| Interpretation† | 20 | Interpretation of the results, taking into account study hypotheses, sources of potential bias or imprecision, and the dangers associated with multiplicity of analyses and outcomes | In addition, take into account the choice of the comparator, lack of or partial blinding, and unequal expertise of care providers or centers in each group | 13,14 |
| Generalizability† | 21 | Generalizability (external validity) of the trial findings | Generalizability (external validity) of the trial findings according to the intervention, comparators, patients, and care providers and centers involved in the trial | 14 |
| Overall evidence | 22 | General interpretation of the results in the context of current evidence |  | 15 |

*Additions or modifications to the CONSORT checklist. CONSORT = Consolidated Standards of Reporting Trials.

†This item was modified in the 2007 revised version of the CONSORT checklist.
