# Supplementary figures and images for: Nutritional counseling with or without mobile health technology: a randomized open-label standard-of-care-controlled trial in ALS
Source: BMC Neurol. 2019 May 29;19:104. doi: 10.1186/s12883-019-1330-6 (PMC6540456; doi:10.1186/s12883-019-1330-6)

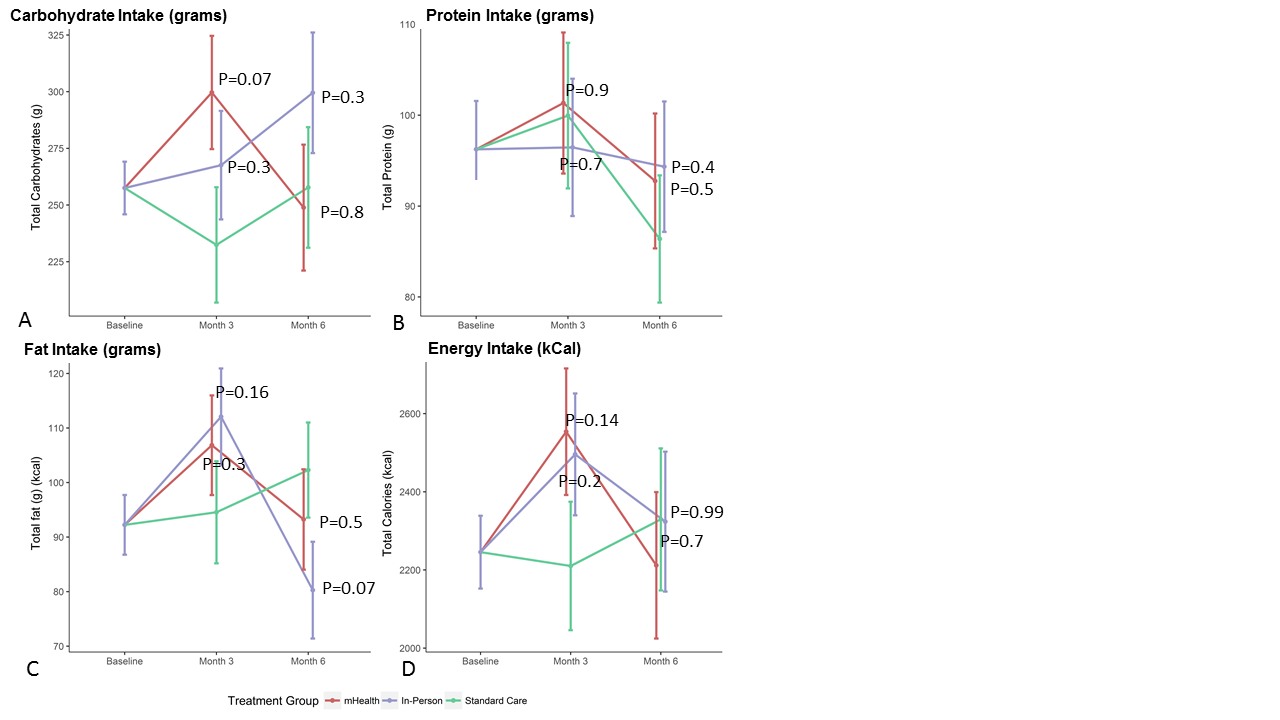

Supplement: Supplementary file 2 — Figure S1. Change from baseline in macronutrient intake using 24-h recall data. Supplementary Figure S1A. Change from baseline in total carbohydrate intake in grams. Supplementary Figure S1B. Change from baseline in total protein intake in grams. Supplementary Figure S1C. Change from baseline in total fat intake in grams. Supplementary Figure S1D. Change from baseline in total calories (in kCal). Red = mHealth; Blue = In-person dietary counseling; Green = Standard Care. Error bars represent 1 standard error around the mean. (TIF 174 kb) [file 12883_2019_1330_MOESM2_ESM.tif]

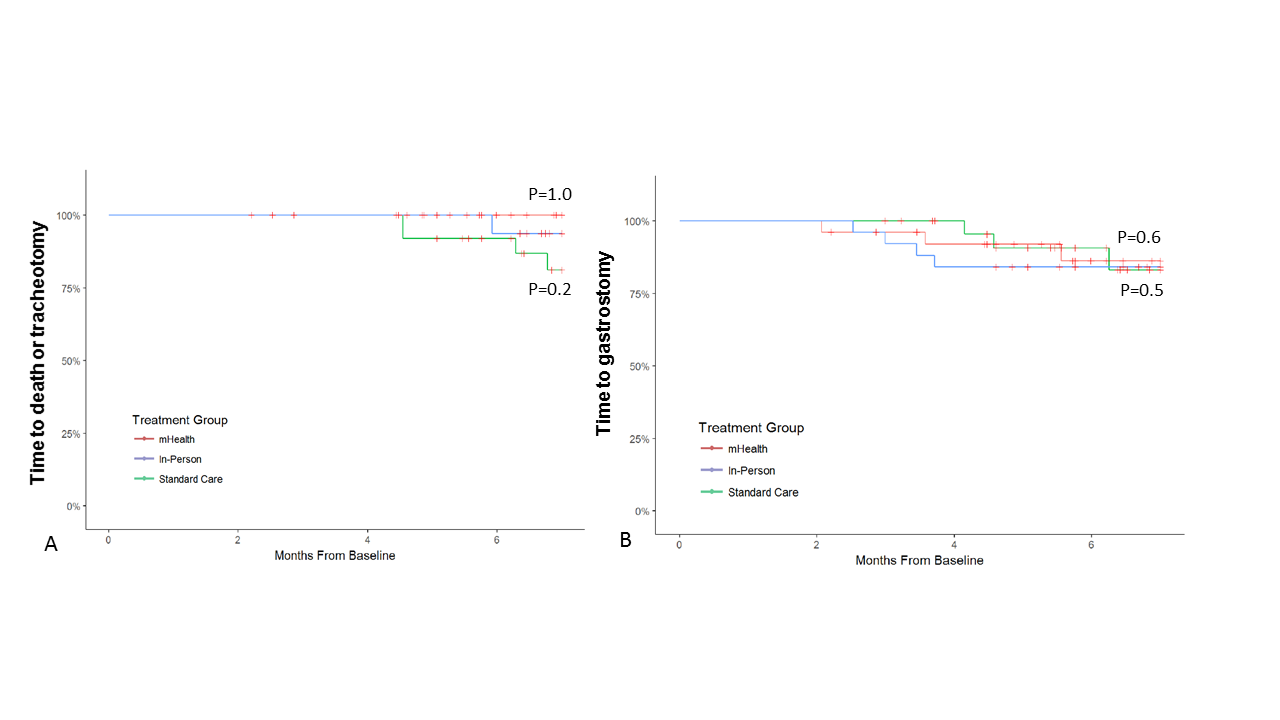

Supplement: Supplementary file 3 — Figure S2A. Kaplan-Meier survival curves for time to death, permanent assisted ventilation or tracheotomy by treatment group. Figure S2B. Time to gastrostomy by treatment group. Red = mHealth; Blue = In-person dietary counseling; Green = Standard Care. The log-rank test result for the difference in survival p = 1·0 for the difference between the mHealth group and the standard care group, and p = 0·2 for the difference between the in-person counseling group and the standard care group. (TIF 99 kb) [file 12883_2019_1330_MOESM3_ESM.tif]

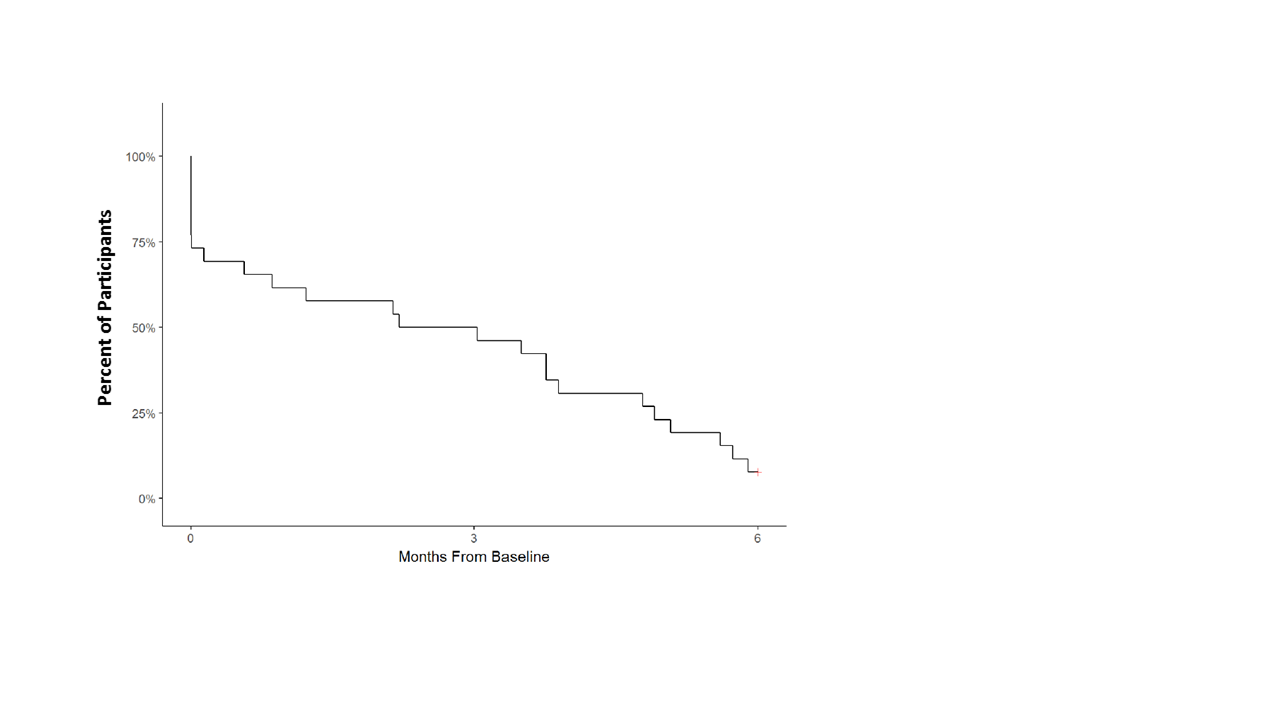

Supplement: Supplementary file 4 — Figure S3. Kaplan-Meier curve for time to last data entry into the mHealth app. Data entry time points were extracted from the mHealth app and used to create a survival curve. (TIF 63 kb) [file 12883_2019_1330_MOESM4_ESM.tif]
